# New Pharmacopœia

**Published:** 1814-07

**Authors:** Richard Walker

**Affiliations:** Oxford


### *For the Medical and Physical Journal.*

### *New Pharmacopœia; by* Mr. Richard Walker, of Oxford.

THE following remarks, although but lately committed to paper, presented themselves to me on looking over the last or present London Pharmacopœia, published in 1809. I offer them to public notice with due deference to the high authority from whence the subject that caused them came, and likewise with much diffidence on my own part, cherishing a hope, however, that at least some of these observations, particularly those respecting *weights and measures*, and likewise the *table of doses for different ages*, which I have constructed, may be found not unworthy of attention.

*Oxford,* RICHARD WALKER.
*May* 9, 1814.

---

The indispensable necessity of applying *new names* to *old medicines* successively, in consequence of the progressive improvement in chemistry, has been a source of considerable inconvenience to the scientific practitioner, and of great perplexity and error amongst compounders of medicines, whose want of sufficient knowledge in chemistry might unqualify them for readily reverting to the chemical elements of which a medicine is composed; and whose deficiency in education, if any such there be, may not have prepared them for comprehending the *rationale* of the names assigned to certain articles founded on their chemical composition.

It were to have been wished that in the present improved, or rather perfect and settled state of medical chemistry, the New London Pharmacopœia of 1809 would have exhibited names, in all instances, perfectly indicative of the chemical compositions to which they were assigned, and consequently have afforded some chance of their remaining permanent or fixed.

It is to be regretted, however, that among other reasons, it has been found necessary to keep in view the probable errors which might arise from ignorance or inattention in the compounders of medicines: hence, in several instances where it is allowed it might have been done, it has been judged prudent to depart from this principle, lest a too great similarity of names in certain chemical medicines differing so widely in their qualities as to produce very serious consequences, if mistaken one for the other, should ensue from it in practice.

Thus it is declared that hydrargyri submurias, the new name assigned to what was formerly called *calomel*, should, according to its chemical elements, have been denominated hydrargyrus hydrargyrus muriatus; but this has been dispensed with, lest in practice it should be confounded with the hydrargyrus muriatus (corrosive sublimate) of the preceding Pharmacopœia; and it is likewise admitted that the term hydrargyri oxymurias is not strictly applicable to the substance formerly known by the name of *corrosive sublimate*.

The exact component or constituent principles of these two mercurial salts, in every particular, seems, even at this time, not clearly defined; but enough, I apprehend, is known respecting them in order to assign a correct, permanent, and distinctive name to each, viz. that the latter (corrosive sublimate) is an oxydated muriate of mercury, and the other (calomel) relatively to that, a sub-oxydated muriate of mercury.

Moreover, the ammonia præparata of the Pharmacopœia of 1787, is, in the present Pharmacopœia, denominated indiscriminately, in some places, carbonate of ammonia, and in others, subcarbonate of ammonia; it is true, that this salt is not ordinarily obtained in a perfectly neutralised state, but one name or other should be adhered to; the former, perhaps, with most propriety, especially as there is but one preparation of it.

To these observations I shall venture to add one more, which is, that distinctions in names, arising from a difference in colour, as occur particularly in some of the preparations of mercury, appear too unscientific to be adopted in a work of such importance; and that it might be desirable if appropriate names, sufficiently distinctive, could be applied, independently of this trite or common-place mode of distinction.

It is impossible, with the utmost caution, to prevent the errors of ignorance or inattention, and which we know have ever happened occasionally, and which, indeed, can be obviated only by the exclusion of unqualified persons, viz. by admitting such persons only as pharmaceutical compounders, who have been duly initiated in that department, and who, consequently, are competent to initiate others; but, however, it should be observed, that the precaution of giving the synonyms of the former with the present Pharmacopœia, would be sufficient to guard against any mistakes whatever, excepting such as might arise from the most gross inattention. Every regular-bred apothecary knows well, that there is much more required in a pharmaceutical compounder than the mere knowledge of the articles and quantities.*

With

* Some years ago I accidentally entered into conversation respecting

With respect to the new Pharmacopœia itself, at least so far as relates to the propriety of the formulæ, presuming it to have been produced by the united efforts of persons best qualified for such an undertaking, notwithstanding any fancied improvements in a few instances which might present themselves to myself, or be suggested by others, I can readily believe that were we possessed of all the reasons which influenced those that framed the formulæ, we should have very little reason, if any, for complaint.

It is proper to observe here, moreover, that we are indebted to the authors of the new Pharmacopœia for many improved formulæ of efficacious old medicines, and for the introduction of several new ones.

It was a matter of considerable satisfaction to me, on the appearance of the new New London Pharmacopœia, to find that so many articles which had retained a place in the materia medica of the preceding Pharmacopœia, had been expunged from the new one of 1809; and that the whole of these articles, including a few others, were such as I had, from the most wearisome experience of their inefficacy, omitted in a "Table of efficacious Medicines," which table was formed several years before the new Pharmacopœia was expected.

This satisfaction did not arise so much from any greater confidence it might have afforded me in the opinions I had formed of these articles, but chiefly from the evidence it presented to me of the improvement respecting the true knowledge of the efficacy of medicines, and the prospect of its future improvement, to which, I flatter myself, I may be in some degree instrumental.

With respect to the new articles introduced into the present Pharmacopœia, I am pleased to see so few of the numerous articles which have been recommended for trial in practice, and ignorantly puffed up as medicines of extraordinary efficacy.

The only fresh articles introduced into the materia medica, indeed, deserving in my opinion of notice, or which have any claim to the title of remedy, during my acquaintance ing pharmacy, &c. with a very respectable druggist, who prepared large quantities of pharmaceutical compositions for sale, and likewise compounded medical prescriptions, when, to my great surprise, I found that in all instances he used *avoirdupois weights* (the small ones excepted, viz. from the drachm downwards) instead of *troy-weights*, appearing to be totally unconscious or regardless of the difference! and it was not without some difficulty that I convinced him of his error, and the consequences resulting from it.

with the profession of physic, which is now a period of nearly forty years, are kino, folia digitalis, and arsenicum.

The first and second of these articles were first introduced into public or general notice, or rather sanctioned in practice, through the medium of the Pharmacopœia of 1787; and the latter, although a familiar remedy for many years under the formulæ of Fowler's drops, forms a new article in the present Pharmacopœia of 1809.*

Of these, kino, which in my opinion entirely supersedes catechu as a remedy in the same intention, viz. as an astringent in alvine fluxes, &c. is alone deserving, in my opinion, of unqualified commendation, and is, unquestionably, a most valuable acquisition to the materia medica, affording that efficacy which is not to be obtained by any other medicine in the same intention.

The folia digitalis and arsenicum are certainly efficacious remedies, but are each of such insidiously-deleterious qualities, the latter especially, as almost to deter practitioners from administering them.

Although there was no formula of a tincture for either kino or folia digitalis in the Pharmacopœia of 1787, we have, for many years, possessed a formula for each in practice.

I have witnessed, it is true, considerable disapprobation expressed, respecting several of the *newly-modified formulæ*, in the Pharmacopœia of 1809, particularly in the instance of the *confectio aromatica*. I venture to believe, however, that I can readily account for this complaint, and remove the cause of it. I suspect, that some pharmaceutical compounders may not be aware of the superior quality of what is ordinarily called *hay-saffron*, above that which is called *cake-saffron*, and might, perhaps, be tempted to use the latter, in consequence of its being a cheaper article.

I am authorised by experience to say, that if hay-saffron be used, in the proportion mentioned in the new Pharmacopœia, carefully dried and finely pulverized, there will be no cause for dissatisfaction; in fact, my old method of making this confection approximated in some degree to the

* The *digitalis* is not a new remedy, a decoction of the root of the same plant having, for many years before, been in use as a powerful remedy in dropsy: the leaf, however, is far superior, used discretionally, in the same intention. The *folia digitalis*, moreover, is a most powerful sedative; consequently, judiciously administered, useful in some hæmorrhages, and in other instances, where the system requires to be suddenly lowered.

one one now presented to us. Of the six ounces of saffron, directed in the old Pharmacopœia, I used three ounces of cake-saffron, with the zedoary, for the infusion directed, and the other half, viz. three ounces, by weight, of hay-saffron, in its ordinary state, afterwards dried and reduced to powder; the sugar, however, I previously dissolved in the infusion, in the manner of a syrup, and added warm to the other ingredients.

I must confess I cannot help feeling a predilection for the old composition, managed according to the manner I have mentioned; possibly the effect of prejudice alone, arising from habit, believing the zedoary, which is now omitted, communicated a peculiarly aromatic flavour, as well as richness of colour, to the composition.

N. B. The ordinary method pursued in mixing together powders and liquids, is to add the powder to the liquid; but I think it may be considered as a general rule, that where the liquid is of a thin consistence, it is, in all instances, best to add the liquid to the powder; observing, however, that just so much of the liquid be added at first as will admit of the powder and liquid being thoroughly incorporated, adding the remainder by degrees. I have even found that in making cerates, unguents, &c. into the composition of which powders enter, I can produce a more uniform homogeneous incorporation, provided the liquid be sufficiently hot and thin, as where wax and oil are melted together, than by any other method; which not unfrequently leaves the powder, as prepared calamine, or white lead, in minute clots, notwithstanding the most attentive stirring.

I cannot, however, forbear expressing my disappointment in finding an old remedy, which has constantly preserved a place in the various Pharmacopœias, under different modifications, viz. the pulvis basilicus of former Dispensatories, and pulvis scammonii and calomel of the last, viz. of 1787, omitted in the present, without even the appearance of a substitute for it. But since, as I have remarked, it has been the fate of several old medicines to be expunged from the Dispensatory at one time and to be restored again in a succeeding one, (a most striking and humiliating proof of the instability of medical knowledge,) it may be no matter of surprise if this old favourite should be restored again to its place in the next revisal of the London Pharmacopœia.

Would there not have been as much propriety in retaining this composition in the present Pharmacopœia, either according to the formula of 1787, or a better one, if it could have have been hit upon, under the name of pulvis hydrargyri submuriatis, or some such name, as in the introduction of Plummer's pill, (unquestionably an excellent medicine,) under the name of pilulæ hydrargyri submuriatis?

Thus, in the present Pharmacopœia, we have an instance of a useful old medicine, which was discarded in the Pharmacopœia of 1787, viz. the elixir vitrioli dulce, of the P. L. for 1745, being restored again in the present Pharmacopœia, under the name of spiritus ætheris aromaticus; and, on the other hand, a medicine which, in my opinion, has justly been discarded years ago, viz. sal enixum, which now reappears under the name of potassæ supersulphas.

Perhaps it is to be regretted, that the task of preparing the English edition had not been undertaken by a professional gentleman, who, with equal competency to the gentleman who has executed it, might have possessed more leisure; since, unquestionably, the most perfect edition of this work is not entirely free from inaccuracies, evidently the effect of haste or oversight.

Of these, I shall take the liberty of noticing one, which to persons commencing the practice of physic might tend to mislead them.

The instance I allude to is the Nosological Table, (substituting the *minim* for the *drop*,) where, notwithstanding the author's precaution on this point, in a different part of the work, the minim in measure is evidently confounded with the drop; arising, apparently, from almost implicitly copying the Table of the preceding Pharmacopœia of 1787, in which the drop, in the way of measuring liquids, was erroneously considered as equivalent to the grain by weight, in solids, viz. the sixtieth part of a drachm; thus we find the dose of tinct. opii stated to be from ♏ x to ʒſs in the new Pharmacopœia, and from ℈ſs to ℈ij in the old one, the preparation being the same in each; whereas the dose of opium itself, in both works, is more properly stated to be from half a grain upwards.

The error respecting the drop being considered as the sixtieth part of a drachm in measure, has, I have no doubt, been productive occasionally of disagreeable consequences in dispensing medicines of an active nature, as tinct. opii, &c. in large quantities at a time, as is sometimes done for patients living at a distance, where measuring has been substituted for dropping, without making due allowance for the difference.

It is remarkable, that, in the new Pharmacopœia, common pitch and Burgundy pitch are both designated under the name name of pix arida; viz. in the unguentum picis aridæ, and emplastrum picis compositum; this inaccuracy, if I do not err in considering it such, is probably attributable to the same cause.

Such inaccuracies as are to be found in this work are not to be wondered at in a publication embracing such a variety of intricate matter, by a professional gentleman whose time and mind are much occupied in the practice of his profession; and will probably be considered by every candid examiner, as proceeding from unavoidable haste in the execution of it.

Weights and Measures.—The new method directed for measuring minute portions of liquids by the minim measure, instead of by drops, reminds me of a circumstance which occurred to me long ago.

Considering the method of dropping, and measuring liquids, especially with measures of a considerable diameter, as very vague and indefinite, and that perfect accuracy was to be obtained only by weighing liquids as well as solids—a ready way occurred to me of effecting it, viz. by obtaining scales of the various sizes required, differing only in the circumstance of one scale consisting of a glass cup, with a pouring lip, and forming an exact counterpoise to the other scale, made in the ordinary way.

Weighing liquids, I am certain, is the only method to be relied on for obtaining accuracy sufficient for nice chemical combinations. It is scarcely necessary to add, that in all instances the measure should be rinced, as it were, with the liquid which forms the vehicle. It seems to be the prevailing opinion, that the minim measure is not likely to supersede the old method of dropping liquids in practice.

The chief advantage of drop-measuring is, that the smallest portion of any liquid may be obtained without loss; hence its peculiar application in the use of essential oils.

The relative proportions between the minim and the drop, in the various liquids used in medical practice, may be appreciated, taking water as a standard of comparison, in a general way, thus:

| | | |
|---|---|---|
| Water ---------- | gutt. i. | equal to ♏i. |
| Proof Spirit ------ | gutt. ii. | |
| Alcohol -------- | gutt. iii. | |

It is a fact commonly known, that the bulk of the drop is affected by a difference in the size of the vial or bottle used, and even by the greater or less thickness in its lip; such such a vial, therefore, should be chosen, which may readily be done, as will cause a drop of water to tally exactly with a minim, or rather any assumed number of drops, viz. 60, for instance.

Without noticing the various inconveniences and difficulties which must necessarily attend the general application of the minim measure, I shall merely mention one which appears to me as an insuperable objection to its general use, and which seems to have been entirely forgotten or overlooked, viz. that the whole of the liquid contained in the tube measure, (the minim measure to which I chiefly allude here,) cannot be obtained without rincing, or agitating the tube in the vehicle, or mixture; the necessary consequence of which will be a superaddition of the liquid adhering to the outside of the tube: this, in so small a quantity as five drops, the extent of this measure, is not unworthy of notice in any liquid, and in the instances of essential oils, concentrated mineral acids, &c. is considerable indeed.

The propriety of reducing liquids by a regular and exact gradation from the largest to the smallest quantities, is unquestionably a great desideratum; and it appears to me to be obtainable only, as in the instance of solids, by weight, and not by measure; and this, I flatter myself, as I have shown above, is not impracticable. Moreover, were this mode adopted, and the specific gravities of liquids, both in a simple and impregnated state, ascertained, we should obtain the precise degree of impregnation in every instance.

At the commencement, or rather in the prosecution of my philosophical and chemical experiments, I soon found the necessity, on account of convenience as well as accuracy, of weighing liquids of every kind; and by this means alone, it has often occurred to me, that error and uncertainty can be banished from pharmacy. Moreover, I might mention another circumstance, viz. the difference in space liquids occupy, at a comparatively cold or warm temperature; which is peculiarly the case in such liquids as by natural cold approximate to a solid state, as certain oils, &c. even this circumstance is of consequence in making cerates, &c.

The ardour for investigation in any person prompted to discover new facts, leads him naturally to a degree of precision unknown to others not thus influenced; and precision, especially when, at the same time, it includes a greater degree of convenience, is particularly desirable.

 A TABLE,

A TABLE, shewing the Proportion of Doses, according to the Age of the Person; the full Dose being one Scruple, one Drachm, or one Ounce.

| | | | | | | | | | |
|---|---|---|---|---|---|---|---|---|---|
| Years 30 | ℈j | ʒj | ℥j | | Years 13 | gs. $9\frac{1}{3}$ | gs. 28 | ʒ3 | gs. 44 |
| 29 | gs. $19\frac{2}{3}$ | gs. 59 | ʒ7 | gs. 52 | 12 | $8\frac{2}{3}$ | 26 | 3 | 28 |
| 28 | $19\frac{1}{3}$ | 58 | 7 | 44 | 11 | 8 | 24 | 3 | 12 |
| 27 | 19 | 57 | 7 | 36 | 10 | $7\frac{1}{3}$ | 22 | 2 | 56 |
| 26 | $18\frac{2}{3}$ | 56 | 7 | 28 | 9 | $6\frac{2}{3}$ | 20 | 2 | 40 |
| 25 | 18 | 54 | 7 | 12 | 8 | 6 | 18 | 2 | 24 |
| 24 | $17\frac{1}{3}$ | 52 | 6 | 56 | 7 | $5\frac{1}{3}$ | 16 | 2 | 8 |
| 23 | $16\frac{2}{3}$ | 50 | 6 | 40 | 6 | $4\frac{2}{3}$ | 14 | 1 | 52 |
| 22 | 16 | 48 | 6 | 24 | 5 | 4 | 12 | 1 | 36 |
| 21 | $15\frac{1}{3}$ | 46 | 6 | 8 | 4 | $3\frac{1}{3}$ | 10 | 1 | 20 |
| 20 | $14\frac{2}{3}$ | 44 | 5 | 52 | 3 | $2\frac{2}{3}$ | 8 | 1 | 4 |
| 19 | 14 | 42 | 5 | 36 | 2 | 2 | 6 | | 48 |
| 18 | $13\frac{1}{3}$ | 40 | 5 | 20 | 1 | $1\frac{1}{3}$ | 4 | | 32 |
| 17 | $12\frac{1}{3}$ | 37 | 4 | 56 | Months 9 | 1 | 3 | | 24 |
| 16 | $11\frac{1}{3}$ | 34 | 4 | 32 | 6 | $\frac{2}{3}$ | 2 | | 16 |
| 15 | $10\frac{2}{3}$ | 32 | 4 | 16 | 3 | $\frac{1}{3}$ | 1 | | 8 |
| 14 | 10 | 30 | 4 | | 1 | $\frac{1}{6}$ | $\frac{1}{2}$ | | 4 |

N. B. The doses are regulated according to an ordinary habit of body, respecting strength; therefore the dose may be somewhat increased or diminished, as circumstances may require.

For females a deduction may be made, according to circumstances, of about one-fifth or one-fourth part.

It will be apparent, that the construction of this Table demanded a degree of minuteness which in practice may be discretionally dispensed with.

The age of 40 may be considered, in strength of constitution, as equal to 30;—50, to 25;—60, to 20;—70, to 16;—and 80, to about 13;—and ordinarily requiring similar doses, varying according to the difference in debility or strength at those ages. Men possessing naturally a good vigorous constitution, and are careful in preserving it, do not arrive perfectly to their acme until they have reached the age of 35; but 30 may be considered as a mature age, sufficient to regulate a table of doses of medicines.

